# Supplementary material for: Modelling Skylarks (Alauda arvensis) to Predict Impacts of Changes in Land Management and Policy: Development and Testing of an Agent-Based Model
Source: PLoS One. 2013 Jun 6;8(6):e65803. doi: 10.1371/journal.pone.0065803 (PMC3675089; doi:10.1371/journal.pone.0065803)
Supplement: Supporting Information S4 — The skylark ODdox as a zipped archive. (ZIP) [file pone.0065803.s004.zip › Skylark_ODdox/functions_0x75.html]

ALMaSS Skylark ODdox: Class Members


ALMaSS Skylark ODdox
 2.0


- Main Page
- Related Pages
- Classes
- Files

- Class List
- Class Index
- Class Hierarchy
- Class Members

- All
- Functions
- Variables
- Typedefs

- a
- b
- c
- d
- e
- f
- g
- h
- i
- j
- k
- l
- m
- n
- o
- p
- r
- s
- t
- u
- v
- w
- x
- y
- z
- ~

Here is a list of all class members with links to the classes they belong to:

### - u -

- UMarginTest()
  : Landscape
- UnsprayedFieldMargin()
  : UnsprayedFieldMargin
- UnsprayedMarginAdd()
  : Landscape
- UnsprayedMarginScan()
  : Landscape
- UpdateQuality()
  : SkTerritories
- Urban()
  : Urban
- UrbanNoVeg()
  : UrbanNoVeg
- UrbanPark()
  : UrbanPark
- UserDefinedFarm()
  : UserDefinedFarm
- UserDefinedFarm1()
  : UserDefinedFarm1
- UserDefinedFarm10()
  : UserDefinedFarm10
- UserDefinedFarm11()
  : UserDefinedFarm11
- UserDefinedFarm12()
  : UserDefinedFarm12
- UserDefinedFarm13()
  : UserDefinedFarm13
- UserDefinedFarm14()
  : UserDefinedFarm14
- UserDefinedFarm15()
  : UserDefinedFarm15
- UserDefinedFarm16()
  : UserDefinedFarm16
- UserDefinedFarm17()
  : UserDefinedFarm17
- UserDefinedFarm18()
  : UserDefinedFarm18
- UserDefinedFarm19()
  : UserDefinedFarm19
- UserDefinedFarm2()
  : UserDefinedFarm2
- UserDefinedFarm20()
  : UserDefinedFarm20
- UserDefinedFarm21()
  : UserDefinedFarm21
- UserDefinedFarm22()
  : UserDefinedFarm22
- UserDefinedFarm23()
  : UserDefinedFarm23
- UserDefinedFarm24()
  : UserDefinedFarm24
- UserDefinedFarm25()
  : UserDefinedFarm25
- UserDefinedFarm26()
  : UserDefinedFarm26
- UserDefinedFarm27()
  : UserDefinedFarm27
- UserDefinedFarm28()
  : UserDefinedFarm28
- UserDefinedFarm29()
  : UserDefinedFarm29
- UserDefinedFarm3()
  : UserDefinedFarm3
- UserDefinedFarm30()
  : UserDefinedFarm30
- UserDefinedFarm31()
  : UserDefinedFarm31
- UserDefinedFarm32()
  : UserDefinedFarm32
- UserDefinedFarm33()
  : UserDefinedFarm33
- UserDefinedFarm34()
  : UserDefinedFarm34
- UserDefinedFarm35()
  : UserDefinedFarm35
- UserDefinedFarm36()
  : UserDefinedFarm36
- UserDefinedFarm4()
  : UserDefinedFarm4
- UserDefinedFarm5()
  : UserDefinedFarm5
- UserDefinedFarm6()
  : UserDefinedFarm6
- UserDefinedFarm7()
  : UserDefinedFarm7
- UserDefinedFarm8()
  : UserDefinedFarm8
- UserDefinedFarm9()
  : UserDefinedFarm9


- Generated on Thu Jan 10 2013 13:15:39 for ALMaSS Skylark ODdox by
   1.8.1.1
